# Why don’t urban youth in Zambia use condoms? The influence of gender and marriage on non-use of male condoms among young adults

**DOI:** 10.1371/journal.pone.0172062

**Published:** 2017-03-23

**Authors:** Jessie Pinchoff, Christopher B. Boyer, Namuunda Mutombo, Rachna Nag Chowdhuri, Thoai D. Ngo

**Affiliations:** 1 Research Department, Innovations for Poverty Action, New Haven, Connecticut, United States of America; 2 Society for Family Health, Lusaka, Zambia; 3 Innovations for Poverty Action Zambia, Jesmondine, Lusaka, Zambia; 4 Poverty, Gender and Youth Program, Population Council, One Dag Hammarskjold Plaza, New York, New York, United States of America; International AIDS Vaccine Initiative, UNITED STATES

## Abstract

**Background:**

Zambia experiences high unmet need for family planning and high rates of HIV, particularly among youth. While male condoms are widely available and 95% of adults have heard of them, self-reported use in the past 12 months is low among young adults (45%). This study describes factors associated with non-use of male condoms among urban young adults in Zambia.

**Methods:**

A household cross-sectional survey in four urban districts was conducted from November 2015 to January 2016 among sexually active young adults ages 18–24 years. A random walk strategy was implemented in urban areas; eligible, enrolled participants were administered a survey on household characteristics, health access, and knowledge, attitudes and practices related to contraception. Relative risk regression models were built to determine factors associated with the decision to not use a male condom (non-use) at most recent sexual intercourse.

**Results:**

A total of 2,388 individuals were interviewed; 69% were female, 35% were married, and average lifetime sex partners was 3.45 (SD±6.15). Non-use of male condoms was 59% at most recent sexual intercourse. In a multivariate model, women were more likely to report non-use of a male condom compared with men (aRR = 1.24 [95% CI: 1.11, 1.38]), married individuals were more likely to report non-use compared with unmarried individuals (aRR = 1.59 [1.46, 1.73]), and those residing in the highest poverty wards were more likely to report non-use compared with those in the lowest poverty wards (aRR = 1.31 [1.16, 1.48]). Those with more negative perceptions of male condom use were 6% more likely to report non-use (aRR = 1.06 [1.03, 1.09]). Discussion regarding contraception with a partner decreased non-use 13% (aRR = 0.87 [0.80, 0.95]) and agreement regarding male condom use with a partner decreased non-use 16% (aRR = 0.84 [0.77, 0.91)]).

**Discussion:**

Non-use of male condoms is high among young, married adults, particularly women, who may be interested in contraception for family planning but remain at risk of STI infection. Effective marketing strategy of dual protection methods to this population is critical.

## Background

Globally, 220 million women experience an unmet need for family planning (FP). [1] Expanded access to FP services in sub-Saharan Africa would result in a projected two thirds reductions in unintended pregnancies, a three quarters reduction in unsafe abortions, a 69% decrease in maternal deaths and a 57% decrease in newborn deaths. [2] In addition, women in sub-Saharan Africa are at increased risk of sexually transmitted infections (STIs) including HIV; young women ages 15–24 years old are twice as likely to be living with HIV compared with young men. [3–5] To ensure protection against both unintended pregnancy and HIV/STI infection, public health programs should emphasize dual protection; dual protection refers to either promotion of barrier methods (such as male or female condoms) for both pregnancy and HIV/STI prevention, or modern contraception coupled with condom use. [4] Male condoms serve as a cornerstone of family planning and HIV/STI prevention programs; despite widespread availability and knowledge of this method, barriers to consistent use remain, particularly among young adults.

Urban Zambia is an important context for the development of effective condom promotion strategies. Zambia has a high rate of unmet need for FP (27%), and its capital city of Lusaka has high rates of poverty and high HIV prevalence of 19.4%). [6] Urban women in Lusaka are more likely to use a FP method than rural women, reflecting wider availability and easier access in urban areas, in addition to social factors. The Zambian government has demonstrated a strong commitment to expanding FP services. [7] However, consistent condom use overall is low: the 2013–14 Demographic and Health Survey (DHS) reported male condom use among young adults (15–24 years) at high-risk sex (intercourse with a non-marital, non-cohabitating partner) in the past 12 months was only 45.1%. [8]

There are myriad social and structural barriers to non-use of condoms in Zambia (and other sub-Saharan African settings), including condom stock outs, stigma around promiscuity and condom use, religion, and lack of knowledge regarding condom use. [9–13] An analysis of the 2009 Zambia Sexual Behaviour Survey data found that more than two-thirds of young adults and adolescents agreed that condoms promoted promiscuity, a pervasive belief in many regions including sub-Saharan Africa. [9] Linked to this, condom use is often the most consistent with partners before or outside of marriage; once a committed relationship is established, condom use decreases. However, due to the high prevalence of HIV/STI’s and multiple and concurrent partnerships, condom use among married partners may continue to be recommended, particularly for serodiscordant couples. [14] However, negotiating condom use in a marriage is more difficult due to trust issues and the implication that a partner is being unfaithful. [15–18] Gender inequality also plays a major role; men are often considered the sexual decision-makers regarding condom use and women may not be able or willing to consistently impose strategies for protection. [19]

There have been some studies documenting various factors that result in non-use of male condoms, but few focus specifically on young adults (18–24 years) living in urban centers in sub-Saharan Africa. With the high unmet need for FP and high HIV prevalence in urban Zambia, and growing young adult population in the region, it is critical to systematically and rigorously measure factors associated with non-use of male condoms so that investment in FP and STIs/HIV prevention programs is strategic and evidence-based. This paper investigates demographic characteristics, sexual behaviors and contraceptive knowledge, access and choices on non-use of male condoms among more than 2,000 urban young individuals in Zambia.

## Methods

### Study population and survey

A cross-sectional survey of young individuals (aged 18–24 years) living in 40 urban wards (administrative units) in Zambia was conducted between November 2015 and January 2016. This survey was conducted as a baseline survey for a larger impact evaluation of a female condom intervention (AEA ID: AEARCTR-0000899). The population of the city of Lusaka is about 3 million; 40 urban wards from the districts of Lusaka (31 wards), Chilanga (3 wards), Kafue (3 wards) and Chongwe (3 wards) comprise the study area. These 40 wards were included in the survey, and each ward is divided into census enumeration areas.

The geographic centroid was calculated for each census enumeration area in the 40 wards using ArcGIS v10.2 (ESRI, Redlands, CA). In each ward, five centroid points were randomly selected (and five additional points to use as back up). Survey teams were directed to these randomly selected centroid points (as the starting point) using GPS devices (Garmin International, Inc., Olathe, KS). Once the survey teams arrived at the centroid points, surveyors approached every other house walking in four separate directions (north, south, east and west) from the start point. If no one was home, the surveyors attempted a second visit. Households were recorded as visited, refused, or no one home (after the two attempts). If someone was present, surveyors entered and introduced the study, and took a roster of all household members present. Inclusion criteria for the study included being 18–24 years of age, residing in that house for at least 6 months, and being sexually active. If more than one household member was eligible, one was randomly selected using SurveyCTO (Dobility Inc, Cambridge MA). All eligible participants that gave verbal and written informed consent were enrolled in the study. Surveyors were trained to conduct the questionnaire in a private setting, usually within the household, where no one else could overhear responses. Participants received 10 kwacha (1 USD) scratch off cards for mobile phone use as compensation for their participation.

The survey administered was approximately 45 minutes long, and asked All participants gave written informed consent and the project was approved by both the Innovations for Poverty Action Institutional Review Board (IRB) and Zambia’s ERES IRB.

### Sample size calculation

Since there were no statistics available on the proportion of non-use among the specific population targeted for this study, this sample size calculation was determined using available estimates from the 2013–14 DHS as a proxy. According to the 2013–14 DHS, male condom use at last high-risk sex in the past year among 15–24 year olds was 45%. [8] From this, we made the assumption that 55% of these did not use a male condom at last sexual intercourse. Based on this figure, with 80% power to detect the proportion of non-use of male condoms (the primary outcome) within 5% of its true value with a 95% confidence interval, we required a minimum sample size of 1,574 individuals.

### Negative perception of male condom use index

During the interview, participants were asked fifteen true or false questions to gauge their perception of male condom use adapted from an existing World Health Organization tool. [20] Questions were based on common perceptions and stereotypes including “male condoms reduce sexual pleasure” and “male condoms are for sex workers”. Responses to these questions were used to construct an index of negative perceptions using multiple correspondence analysis. The resulting index is mean zero and unit standard deviation, with higher values signifying increasingly negative perceptions.

### Multiple imputation

While the overall response rate to the survey was high (98%), a subset of sexual behavior questions had moderate rates of item non-response, due to the sensitive nature of the questions (15 to 20%). To gauge the sensitivity of observed results to item non-response, we imputed 50 random data sets representing the missing data using multiple chained equations [21] and re-estimated our regression models using Stata’s -**mi**- package. [22] The results were pooled using the Rubin method. [23] Though our statistical inference concerning the results was largely unaffected by the inclusion of missing cases, point estimates for particular covariates varied by more than 10%. Therefore, we present the multiple imputation in the results section.

### Statistical analysis

All relevant variables were tabulated by self-reported non-use of a male condom at last sexual intercourse. A figure was created depicting forms of contraception used stratified by self-reported non-use of a male condom at most recent sexual intercourse. We then estimated the relative risk for each exposure-outcome paring using Poisson regression models with robust variance estimates using the “sandwich” operator. [24] Results from these models are presented as risk ratios (RRs) with 95% confidence intervals (CIs). Covariates of interest were then used to construct multivariable regression models via three stages of model building. Age was not included in the models because of the small age range of participants (18–24 years).

After conducting univariate analyses for each characteristic of interest, presented in Stage 1, covariates of interest were added to the model in three additional stages. Stage 2 controlled for demographic questions such as gender, educational attainment, employment status, and marital status. The proportion of households in each ward living below the poverty line according to recent World Bank modeled estimates was also included. [25] Ward level poverty ranged from 1% to 65% with a median of 20%. Age was considered but not included in the models due to the narrow range (18–24 years) and non-significance in bivariate results.

In the Stage 3 model, sexual health variables were added. These included the number of lifetime sex partners, frequency of sexual intercourse (over the past 30 days), number of children, whether the participant was ever tested for an STI, and whether the participant (or their partner) used another form of contraception (in the past 6 months). Other forms of contraception were categorized into none or traditional (such as rhythm method, withdrawal, or lactational amenorrhea), short-term modern methods (such as pills or injectables), and long acting reversible contraception (LARC) or long acting permanent methods (LAPM) (such as an intrauterine device). These were added to assess if non-use of male condoms changed with use of another contraceptive method. Distance to the nearest health facility was calculated in ArcGIS using the GPS coordinates of the households and health facilities. An interaction term was also included between number of lifetime sex partners (per 5 partners) and frequency of sexual intercourse in the past 30 days (per 5 sexual interactions).

The Stage 4 model added variables on contraceptive knowledge, behaviors and attitudes. These include how many forms of contraception the participant is familiar with, whether the participant reports ever discussing contraception with their partner, whether the both partners agree regarding contraception use, and score on the negative perceptions index. The method used to create this perceptions index is described above.

Lastly, the Stage 4 version of the model was run stratified by marital status due to the significance of this characteristic and the major differences in non-use of male condoms between married and un-married individuals. All p-values presented are 2-sided, those significant at the p<0.05 level of significance are denoted with an asterisk.

## Results

### Study population

A total of 8,137 households were approached for enrollment throughout the 40 wards in Lusaka; 2,787 (34%) did not have household members who were eligible for participation, 2,792 (34%) did not have anyone home after two visits, and 161 (2%) had someone home who refused to participate in the study. A total of 2,388 surveys were successfully completed, and the majority of participants (n = 1,646; 68.9%) were female. Of all participants, the mean age was 21.31 years (SD = 1.94). Most participants had completed secondary schooling (n = 1,847; 77.3%), and were unmarried (n = 1,547; 64.8%). While 1,458 (61%) reported they did not work at all, 318 (13%) reported they worked the entire year, 384 (16%) reported seasonal work, and 227 (10%) reported occasional work. A total of 527 (22%) reported they were currently in school. Most participants lived with someone else: parents (26.8%), other family members (32.7%), or a spouse (33%). Almost half (49%) reported they had one or more children.

### Male condom non-use

A total of 359 (15%) of participants reported never using a male condom; while 1,024 (33%) reported non-use in the last six months, and 1,415 (59%) reported non-use at most recent sexual intercourse. Comparing those who reported non-use of a male condom at most recent sexual intercourse to those who reported use, 1,125 (79.5%) vs 521 (53.5%) were female (p<0.001) (Table 1). Those that were employed reported lower male condom non-use (73.9% vs 83%; p<0.001). Those that were married were much less likely to report male condom use (12.7% of married couples did use a male condom vs 50.7% of unmarried couples; p<0.001). Those that did not use a male condom were from higher poverty wards (54.4% in highest poverty wards did not use a male condom; p<0.001). Of those that did use a male condom, 71.4% had discussed contraception with their most recent partner (compared with 67.7% who did not use a male condom; p = 0.054), and of those that did use a male condom 77.5% reported that they and their partner agreed on using contraception (compared with 69.9% of those that did not use a male condom; p<0.001). Those with a more negative perception of male condom use were more likely to report non-use of a male condom (-0.13 vs 0.26; p<0.001).

**Table 1:** Demographic and sexual health characteristics of participants by use and non-use of male condom at last sexual intercourse.

Variable	Level	Non-use of male condom (n = 1415)	Use male condom (n = 973)	P-value
Gender	Male	290 (20.5%)	452 (46.5%)	<0.001*
	Female	1125 (79.5%)	521 (53.5%)	
Age, mean ± standard deviation (SD)		21.34 ± 1.93	21.27 ± 1.95	0.41
Currently in School	No	1156 (81.7%)	705 (72.5%)	<0.001*
	Yes	259 (18.3%)	268 (27.5%)	
Educational Attainment	Primary	388 (27.4%)	132 (13.6%)	<0.001*
	Secondary	899 (63.5%)	627 (64.5%)	
	Higher	128 (9.0%)	213 (21.9%)	
Employment Status	No	1175 (83.0%)	719 (73.9%)	<0.001*
	Yes	240 (17.0%)	254 (26.1%)	
Marital Status	Unmarried	698 (49.3%)	849 (87.3%)	<0.001*
	Married	717 (50.7%)	124 (12.7%)	
Poverty rate (ward)	0–10%	161 (11.4%)	249 (25.6%)	<0.001*
	10–20%	484 (34.2%)	361 (37.1%)	
	20% +	770 (54.4%)	363 (37.3%)	
Age at first sexual intercourse, mean ± SD		17.33 ± 2.33	17.25 ± 2.48	0.41
Number of lifetime sex partners, mean ± SD		2.81 ± 4.64	4.38 ± 7.75	<0.001*
Frequency of intercourse (last 30 days), mean ± SD		4.05 ± 6.33	2.64 ± 5.13	<0.001*
Number of children	None	522 (37.6%)	628 (68.2%)	<0.001*
	1 or more	868 (62.4%)	293 (31.8%)	
Ever tested for a sexually transmitted infection (STI)?	No	265 (18.7%)	227 (23.3%)	0.006*
	Yes	1150 (81.3%)	746 (76.7%)	
Other contraceptives used (ever)	None/Traditional	528 (37.3%)	507 (52.1%)	<0.001*
	Short-term modern Method	713 (50.4%)	390 (40.1%)	
	LARC/LAPM	174 (12.3%)	76 (7.8%)	
Distance to nearest clinic	< 2.5 km	1086 (79.7%)	832 (88.9%)	<0.001*
	≥ 2.5 km	277 (20.3%)	104 (11.1%)	
Number of contraceptive methods known	< 3	509 (36.0%)	398 (40.9%)	0.015*
	≥ 3	906 (64.0%)	575 (59.1%)	
Discussed contraception with most recent partner	No	454 (32.3%)	278 (28.6%)	0.054*
	Yes	950 (67.7%)	693 (71.4%)	
Partner agrees with using contraception	No	415 (30.1%)	215 (22.5%)	<0.001*
	Yes	964 (69.9%)	741 (77.5%)	
Negative perceptions of condom use index, mean ± SD		0.26 ± 1.11	-0.13 ± 0.94	<0.001*

* Denotes statistical significance p<0.05

Individuals who reported non-use of a male condom at most recent sexual intercourse were more likely to report using another modern contraceptive method, ever. These are not mutually exclusive; report of any contraceptive could occur at any time, compared with male condom use specifically at most recent sexual intercourse, and are not necessarily overlapping. Those that reported ever using a modern short-term method were more likely to report non-use of a male condom at most recent sexual intercourse (59.9 vs 49.6%; p<0.001). Those that reported using a long acting reversible contraceptive / long acting or permanent method (LARC/LAPM) method were also more likely to report non-use of a male condom (92.2% vs 87.7%; p<0.001) (Table 1).

### Negative perceptions index

Table 2 below highlights responses to the 15 true/false questions used to generate the male condom negative perceptions index and includes the weight and contribution of each variable in the index. Questions with the highest contribution to the negative perceptions index include “Male condoms are for sex workers” and “If a woman suggested using male condoms to her partner, it would mean she is promiscuous” (Table 2).

**Table 2:** Tabulation of 15 true or false questions regarding male condom non-use and the relative weight and contribution of each to the negative perceptions index.

Question	Response	Didn't use male condom (n = 1415)	Used male condom (n = 973)	P-value^A^	Negative Perceptions of Male Condom Use Index^B^	
					Weight^C^	Contribution^D^
Condoms prevent pregnancy.	False	140 (10.4%)	69 (7.2%)	0.008	1.285	0.009
	True	1201 (89.6%)	888 (92.8%)		-0.135	0.001
Condoms can help prevent STIs and HIV.	False	140 (10.6%)	87 (9.3%)	0.32	1.127	0.008
	True	1185 (89.4%)	852 (90.7%)		-0.14	0.001
A woman should be able to suggest to her partner that they use a condom during sexual intercourse.	False	137 (10.2%)	64 (6.7%)	0.003	1.321	0.01
	True	1208 (89.8%)	893 (93.3%)		-0.147	0.001
A man should be able to suggest to his partner that they use a condom during sexual intercourse.	False	139 (10.3%)	53 (5.6%)	<0.001	0.914	0.004
	True	1217 (89.7%)	896 (94.4%)		-0.082	0
Condoms are more appropriate for unmarried couples than married couples.	False	506 (38.1%)	358 (38.2%)	0.96	-1.144	0.031
	True	823 (61.9%)	579 (61.8%)		0.751	0.02
I feel uncomfortable buying condoms near my home.	False	448 (33.9%)	357 (37.6%)	0.069	-1.075	0.026
	True	875 (66.1%)	593 (62.4%)		0.676	0.017
It is difficult for a woman to ask her partner to use a condom during sexual intercourse	False	790 (58.2%)	575 (61.1%)	0.17	-1.153	0.047
	True	568 (41.8%)	366 (38.9%)		1.726	0.07
If a woman suggested using male condoms to her partner, it would mean she is promiscuous.	False	869 (64.9%)	720 (76.4%)	<0.001	-1.016	0.044
	True	469 (35.1%)	223 (23.6%)		2.573	0.11
Condoms reduce sexual pleasure.	False	462 (41.9%)	450 (51.7%)	<0.001	-1.56	0.068
	True	640 (58.1%)	421 (48.3%)		1.43	0.063
Having sex with a condom is important.	False	168 (12.5%)	34 (3.5%)	<0.001	3.129	0.045
	True	1171 (87.5%)	928 (96.5%)		-0.264	0.004
Male condoms can slip off the man and get permanently lost inside a woman’s body.	False	659 (65.9%)	532 (70.8%)	0.030	-0.687	0.02
	True	341 (34.1%)	219 (29.2%)		1.811	0.053
Male condoms make it more difficult for the man to achieve orgasm.	False	403 (42.1%)	420 (55.0%)	<0.001	-1.561	0.074
	True	555 (57.9%)	344 (45.0%)		1.682	0.08
Male condoms are for sex workers	False	961 (77.3%)	829 (90.7%)	<0.001	-0.68	0.023
	True	282 (22.7%)	85 (9.3%)		4.021	0.138
Before using a male condom, one should check the expiration date on the package.	False	20 (1.5%)	11 (1.2%)	0.58	-1.466	0.002
	True	1314 (98.5%)	944 (98.8%)		0.021	0
One should always use lubricant with the male condom.	False	630 (70.2%)	544 (70.1%)	1.00	-0.373	0.006
	True	268 (29.8%)	232 (29.9%)		0.854	0.013
The male condom can be reused.	False	1324 (98.3%)	946 (98.5%)	0.74	-0.059	0
	True	23 (1.7%)	14 (1.5%)		3.395	0.012
After using the male condom, it should not be disposed of in the toilet, only the trash.	False	326 (24.5%)	200 (21.3%)	0.077	0.028	0
	True	1003 (75.5%)	739 (78.7%)		-0.01	0

Note:

^A^ P-values are from Fisher's Exact Test of equality of proportions by non-use of male condoms at last intercourse.

^B^ Index calculated using the first dimension of a multiple correspondence analysis (MCA) of condom opinions; the first dimension explained 62.2% of the common variance.

^C^ Index weights are first dimension coordinate from MCA; a respondent's final index value is sum of weights of their responses for questions A through Q.

^D^ Percentage contribution measures the weighted influence of each factor on the index.

### Risk factors for non-use

Model building was conducted from Stage 1, which presents unadjusted bivariate models for each variable. For model stages 2 through 4 adjusted relative risk regression analyses were performed to assess the effect of controlling for different variables on use of a male condom at most recent sexual intercourse (coded non-use of male condom = 1, did use = 0). These models highlight how adding variables at each stage influence the model and how the final model (stage 4) was established.

In the final model (Stage 4), several characteristics continued to be associated with increased self-reported non-use of a male condom at most recent sexual intercourse, that were significant from univariate models. Women were associated with a 24% increased report of non-use compared with men (aRR = 1.24; [95% CI 1,11, 1.38]) (Table 3). Being married was associated with a 59% increase in non-use compared with unmarried participants (aRR = 1.59; [1.46, 1.73]). Those residing in the highest poverty wards (≥20% of households in poverty) were associated with a 22% increase in non-use of male condoms compared with those in the lowest poverty wards (aRR = 1.31; [1.16, 1.48]). Those who reported having any children were associated with a 15% increase in report non-use compared with those who had no children (aRR = 1.15; [1.04, 1.26]). Increasing negative perception index score was associated with a 6% increase in non-use of male condoms (aRR = 1.06; [1.03, 1.09]). (Table 3)

**Table 3:** Factors associated with non-use of a male condom at most recent sexual intercourse.

Variable	Level	(1)		(2)		(3)		(4)	
		Crude (Unadjusted)		Demographics		Demographics + Sexual Health		Demographics +Sexual Health + Knowledge, Attitudes. Behaviors	
		Relative Risk (RR)	95% confidence interval (CI)	Adjusted RR (aRR)	95% CI	aRR	95% CI	aRR	95% CI
Sex	Male	1.00	ref.	1.00	ref.	1.00	ref.	1.00	ref.
	Female	1.75*	(1.59–1.92)	1.33*	(1.20–1.48)	1.25*	(1.12–1.40)	1.24*	(1.11–1.38)
Currently in School	No	1.00	ref.	1.00	ref.	1.00	ref.	1.00	ref.
	Yes	0.79*	(0.72–0.87)	1.08	(0.98–1.19)	1.07	(0.97–1.18)	1.07	(0.97–1.18)
Educational Attainment	Primary	1.00	ref.	1.00	ref.	1.00	ref.	1.00	ref.
	Secondary	0.79*	(0.74–0.84)	0.99	(0.93–1.06)	1.00	(0.94–1.06)	1.05	(0.98–1.12)
	Higher	0.50*	(0.44–0.58)	0.79*	(0.67–0.92)	0.81*	(0.69–0.95)	0.89	(0.76–1.05)
Employment Status	No	1.00	ref.	1.00	ref.	1.00	ref.	1.00	ref.
	Yes	0.78*	(0.71–0.86)	0.89*	(0.81–0.98)	0.89*	(0.82–0.98)	0.91*	(0.83–1.00)
Marital Status	Unmarried	1.00	ref.	1.00	ref.	1.00	ref.	1.00	ref.
	Married	1.89*	(1.78–2.01)	1.59*	(1.48–1.71)	1.55*	(1.42–1.69)	1.59*	(1.46–1.73)
Poverty rate (ward)	0%–10%	1.00	ref.	1.00	ref.	1.00	ref.	1.00	ref.
	10%–20%	1.46*	(1.28–1.67)	1.24	ref.	1.23*	(1.08–1.39)	1.22*	(1.08–1.38)
	20% +	1.73*	(1.52–1.96)	1.35*	(1.19–1.52)	1.33*	(1.17–1.50)	1.31*	(1.16–1.48)
Number of lifetime sex partners (per 5 partners)		0.84*	(0.76–0.94)			0.91*	(0.84–0.99)	0.90*	(0.83–0.99)
Frequency of intercourse (last 30 days) (per 5 sexual acts)		1.07*	(1.04–1.11)			0.97	(0.95–1.00)	0.97*	(0.94–1.00)
No. lifetime partners × Freq. of intercourse						1.03	(1.00–1.07)	1.04*	(1.00–1.07)
Number of children	None	1.00	ref.			1.00	ref.	1.00	ref.
	1 or more	1.63*	(1.52–1.75)			1.13*	(1.02–1.25)	1.15*	(1.04–1.26)
Ever tested for a sexually transmitted infection (STI)?	No	1.00	ref.			1.00	ref.	1.00	ref.
	Yes	1.13*	(1.03–1.23)			0.89*	(0.82–0.98)	0.91*	(0.83–0.99)
Other contraceptives used (ever)	None/Traditional	1.00	ref.			1.00	ref.	1.00	ref.
	Short-term modern Method	1.27*	(1.18–1.36)			0.96	(0.88–1.04)	1.01	(0.92–1.10)
	LARC/LAPM^A^	1.36*	(1.23–1.51)			1.01	(0.91–1.12)	1.05	(0.95–1.17)
Distance to nearest clinic	< 2.5 km	1.00	ref.			1.00	ref.	1.00	ref.
	> = 2.5 km	1.27*	(1.18–1.37)			1.04	(0.97–1.12)	1.04	(0.97–1.12)
No. of contraceptive methods known	No	1.00	ref.					1.00	ref.
	Yes	1.09*	(1.02–1.17)					1.03	(0.96–1.09)
Discussed contraception with most recent partner	No	1.00	ref.					1.00	ref.
	Yes	0.93*	(0.87–1.00)					0.87*	(0.80–0.95)
Partner agrees with using contraception	No	1.00	ref.					1.00	ref.
	Yes	0.86*	(0.80–0.92)					0.84*	(0.77–0.91)
Negative perceptions of male condom use index		1.15*	(1.12–1.18)					1.06*	(1.03–1.09)

^A^ LARC = Long acting reversible contraception; LAPM = Long Acting Permanent Method of contraception

* Denotes statistical significance at p<0.05

In the Stage 4 final model, some characteristics in this model were associated with decreased non-use of male condoms. Being employed was associated with a 9% decrease in non-use of male condoms (aRR = 0.91; [0.83, 1.00]). Participants that were ever tested for an STI were associated with a 9% decrease in non-use of male condoms (aRR = 0.91; [0.83, 0.99]). Discussing contraception with a partner was associated with a 13% decrease in non-use of male condoms and similarly agreeing with a partner regarding whether or not to use male condoms was associated with a 16% decrease in non-use (Table 3).

In the stage 4 model, reporting a higher number of lifetime partners and a higher frequency of sexual intercourse in the last 30 days were both associated with lower non-use of male condoms at most recent sexual intercourse. However, there was an interaction between these two variables, reversing the direction of the association and leading to higher non-use of male condoms. The interaction suggests that as the number of lifetime sexual partners increases by 5 partners and the frequency of sexual interactions increases, non-use of male condoms is associated with a 4% increase (aRR = 1.04; 95% CI [1.00, 1.07]) (Table 3). This suggests that taken together, having more lifetime partners and more frequent sexual activity may reflect a group of ‘high-risk’ individuals associated with higher non-use of male condoms.

### The effect of marital status on non-use of male condoms

Several demographic, sexual health, knowledge, attitudes and behavior variables interact with marital status, therefore the Stage 4 model was run stratified by marital status. When restricted to married individuals, having one or more children was associated with 12% decrease in non-use of male condoms (aRR = 0.88; [0.80, 0.96)] (Table 4). Among married individuals, using other contraceptives such as modern short-term or LARC/LAPM methods was associated with increased non-use of male condoms. Many of the characteristics significant in the full model became insignificant when restricted to married individuals.

**Table 4:** Factors (from stage 4 model) associated with non-use of male condoms, stratified by marital status.

		All		married		unmarried	
Variable	Level	risk ratio (aRR)	95% CI	risk ratio (aRR)	95% CI	risk ratio (aRR)	95% CI
Observations		2,388		841		1,547	
Marital Status = 1, Married		1.59*	(1.46–1.74)				
Sex	Male	1	ref.	1	ref.	1	ref.
	Female	1.24*	(1.11–1.38)	1.16	(0.96–1.40)	1.22*	(1.07–1.39)
Currently in School	No	1	ref.	1	ref.	1	ref.
	Yes	1.07	(0.97–1.18)	1.00	(0.89–1.13)	1.09	(0.96–1.23)
Educational Attainment	Primary	1	ref.	1	ref.	1	ref.
	Secondary	1.05	(0.98–1.12)	1.06*	(1.00–1.12)	1.00	(0.86–1.16)
	Higher	0.89	(0.76–1.05)	1.06	(0.89–1.27)	0.86	(0.69–1.09)
Employment Status	No	1	ref.	1	ref.	1	ref.
	Yes	0.91*	(0.83–1.00)	0.98	(0.89–1.07)	0.85*	(0.73–0.99)
Poverty rate (ward)	0%–10%	1	ref.	1	ref.	1	ref.
	10–20%	1.22*	(1.08–1.39)	1.02	(0.90–1.16)	1.30**	(1.09–1.55)
	20% +	1.31*	(1.16–1.48)	1.04	(0.91–1.18)	1.46*	(1.24–1.74)
Number of lifetime sex partners (per 5 partners)		0.90*	(0.83–0.99)	0.94	(0.83–1.06)	0.89*	(0.80–0.99)
Frequency of intercourse (last 30 days) (per 5 sexual acts)		0.97*	(0.94–1.00)	0.99	(0.96–1.02)	0.90*	(0.81–1.00)
No. lifetime partners × Freq. of intercourse		1.04*	(1.00–1.07)	1.02	(0.99–1.06)	1.05*	(1.01–1.09)
Number of children	None	1	ref.			1	ref.
	1 or more	1.15*	(1.04–1.27)	0.88*	(0.80–0.96)	1.23*	(1.08–1.40)
Ever tested for an STI?	No	1	ref.			1	ref.
	Yes	0.91*	(0.83–0.99)	0.98	(0.89–1.07)	0.88*	(0.78–0.99)
Other contraceptives used (ever)	None/Traditional	1	ref.			1	ref.
	Modern Short Term	1.01	(0.93–1.10)	1.13*	(1.01–1.26)	0.97	(0.86–1.10)
	LARC/LAPM Method	1.06	(0.95–1.18)	1.20*	(1.07–1.36)	1.05	(0.85–1.28)
Distance to nearest clinic	< 2.5 km	1	ref.			1	ref.
	>2.5 km	1.01	(0.99–1.02)	0.99	(0.98–1.01)	1.03*	(1.00–1.06)
Discussed contraception with most recent partner	No	1	ref.	1	ref.	1	ref.
	Yes	0.87*	(0.80–0.95)	0.91*	(0.85–0.98)	0.89*	(0.78–1.01)
Partner agrees with using contraception	No	1	ref.	1	ref.	1	ref.
	Yes	0.84*	(0.77–0.91)	1.00	(0.92–1.09)	0.75*	(0.66–0.85)
Negative perception of condom use index		1.06*	(1.03–1.09)	1.05*	(1.02–1.07)	1.08*	(1.02–1.13)

* Denotes statistical significance at p<0.05

However, among unmarried individuals, several characteristics were associated with non-use of male condoms. Among unmarried individuals, women were associated with a 22% increase in non-use (aRR = 1.22; [1.07, 1.39]) (Table 4). Being employed was associated with 15% decrease in non-use (aRR = 0.85; [0.73, 0.99]), while living in the highest poverty wards was associated with a 46% increase in non-use (aRR = 1.46; 1.24, 1.74]). The interaction between increased number of lifetime sex partners and increased frequency of sexual intercourse was associated with a 5% increase in non-use (aRR = 1.05; [1.01, 1.09]) (Table 4). The association with having one or more children was the opposite direction as for married persons; among unmarried individuals having one or more children was associated with a 23% increase in non-use (aRR = 1.23; [1.08, 1.40]). Having discussed use of contraceptives and agreeing with their most recent sex partner on whether or not to use contraceptives was associated with decreased non-use.

The negative perceptions index and discussing contraceptive with the most recent sexual partner were the only two variables from the Stage 4 model that were statistically significant in the same direction in the full model, for married couples only and for unmarried couples only.

## Discussion

In our analysis, over half (59%) of urban, young Zambians reported non-use of male condoms at most recent sexual intercourse. We identified several significant characteristics associated with non-use at most recent sexual intercourse: women, married couples, individuals in high poverty areas, those with children, and those who had negative perceptions about male condoms. Being employed, having ever been tested for an STI, having had discussed contraception with their most recent partner, and agreeing with their partner regarding contraception decreased non-use of male condoms. These findings shifted slightly when stratified by marital status, suggesting different motivations for non-use of male condoms. Among married individuals, having children was associated with a decrease in non-use suggesting male condom use for birth spacing. Among unmarried individuals, women, the unemployed, the urban poor, and those with a combination of more lifetime sex partners and more frequent sexual intercourse were associated with an increase in non-use. Only two variables, discussing contraception with the most recent sexual partner and negative perceptions index score, remain consistently significant between the two groups. These findings highlight that while targeted messages based on marital status may be effective for young adults, there is also opportunity for more general marketing and behavior change programs to change negative perceptions.

This study is a cross-sectional representative sample of urban, sexually active, young adults (ages 18–24 years) in Zambia, with a survey focusing on sexual and reproductive health and contraceptive behavior. The finding that non-use of male condoms was 59% is between the proportions reported by the 2013–14 DHS (55%) and Zambia Sexual Behavior Survey (63%). [8,26] This may be due to the different definitions and age categories used; the DHS estimate is male condom use for high-risk sex in the past 12 months for 15–24 year olds. The Zambia Sexual Behavior Survey is percent of young single people (15–24 years) who used a male condom at last sexual intercourse. This study is the most recently available data, is uniquely focused on young adults (18–24 years), and is the most thorough questionnaire regarding sexual and reproductive health (that is not focused on HIV-infected persons). Our findings support previous research that suggests sexual relations and condom negotiation are strongly associated with gender, marriage and the power dynamic between men and women. [27] In particular, our findings suggest economically vulnerable, unemployed, unmarried women were most likely to report non-use of male condoms; they may have a low sense of empowerment and have been found to be the least able to negotiate safer sex practices such as condom use. [28]

The finding that married young persons are significantly less likely to use condoms is supported by recent literature, [29,30]. Messaging for married couples is often focused on family planning options, but may leave them open to risk of STI/HIV infection. Some studies suggest that male condoms may not be used by women with regular or live-in partners [31], a critical gap, as an estimated 60–80% of HIV-infected women in sub-Saharan Africa have contracted the virus from their husbands. [32] This also has implications for sero-discordant couples, many of whom do not realize that serodiscordance is possible, sometimes leading to failure for one partner to get tested. It is critical they receive joint couples counseling to ensure both understand the implications of serodiscordance, and are able to discuss and agree on condom use. [33] However, use of condoms within a marital relationship is often viewed with apprehension or even hostility, as it suggests one partner is being unfaithful. [18] It also may be viewed negatively because it does not allow skin-to-skin contact. [34]

This analysis found that married persons with a stronger negative perception of male condoms were significantly less likely to use them, but that those with one or more children were more likely to use them, potentially linking condom use with birth spacing for these couples. For young married couples that wish to postpone pregnancy and birth-spacing, dual protection methods such as male condoms should be recommended to prevent against both risks (unplanned pregnancy and STI/HIV infection) in regions with high prevalence of concurrent sexual partnerships and of STI’s/HIV. While this study did not measure concurrent sexual partnership, the Zambian 2013–14 DHS reported a 10% prevalence of multiple sexual partners was reported, and of these 78% were concurrent. [8] The interactions between use of dual contraception or dual protection methods, multiple concurrent sexual partners, and serodiscordance should be explored further.

Among unmarried individuals, many characteristics including gender, employment status, ward level poverty, and reporting both a higher number of lifetime sex partners and more frequent sexual intercourse in the last 30 days were associated with increased non-use of male condoms, highlighting several high-risk characteristics for non-use of condoms. Although overall married couples were more likely to report non-use of condoms, many of these characteristics were not significant when restricted to married persons only, suggesting very different risks and motivations between married and unmarried persons. Only the negative perceptions index persisted in being significant, highlighting an opportunity for public health messages and behavior change campaigns. If the negative perceptions of male condoms can be addressed, use may increase among all young, urban adults regardless of marital status.

There are limitations in this survey. First, almost 70% of respondents were females, likely because the majority of surveys were conducted during the day when men may be more likely to be outside of the home. This may lead to underreporting of condom use if women are more likely to report condom use due to social desirability bias. Although social desirability bias may be a concern regardless, as all participants may report more condom use since this is a known positive behavior. Second, while we asked about use of other types of contraception, it was not possible to ascertain if a participant that reported using a male condom at most recent sexual intercourse was using another form of contraception at the same time. The way the question was framed allowed for participants to report using various forms of contraception, and asked about condom use at most recent sexual intercourse, but is not phrased to ask if use of an additional contraceptive was concurrent. The question will be modified for the end-line survey for the larger randomized evaluation that is being conducted. Lastly, questions regarding condom use were self-reported; however, by asking about most recent sexual intercourse, recall bias was likely reduced. We also had some missing responses due to the sensitive nature of some of the questions, and multiple imputation methods were used to adjust for this.

Our findings may be generalizable to other urban African settings with high STI/HIV prevalence, particularly where the median age of first marriage is relatively low (18.4 years for women), and the total fertility rate (TFR) based on the 2013–14 DHS is 5.3 (lower in urban areas, 3.7 births per woman). [8] In these settings, dual protection methods are critical to prevent both unplanned pregnancy and STI/HIV infection, and particularly young women are at increased risk. In many of these settings, consistent condom use is falling short of uptake goals and targets, and it is not always clear why. Social marketing campaigns in Zambia have made inexpensive condoms widely available, employing strategies such as mass media advertising and peer education campaigns to increase use. [11] There is some evidence that attitudes have shifted, mainly due to the AIDS epidemic, but it is unclear the extent of these changes. [11]

These findings suggest behavior change programs focused on reducing negative perceptions of male condoms, and fostering discussion between sexual partners regarding contraception, will be the most effective for the general population of young, urban adults. Coupling these messages with programs targeted to unmarried persons to increase female empowerment, ability to advocate for safe sex, and reduce risky behavior, may result in increased numbers of protected sexual acts. Separately tailoring messaging campaigns for married couples in this young age category may be useful, as married couples are less likely to use condoms despite continued risk of HIV infection [35], except potentially for birth spacing.
